# Complete Genome Sequences of 44 *Arthrobacter* Phages

**DOI:** 10.1128/genomeA.01474-17

**Published:** 2018-02-01

**Authors:** Karen K. Klyczek, Deborah Jacobs-Sera, Tamarah L. Adair, Sandra D. Adams, Sarah L. Ball, Robert C. Benjamin, J. Alfred Bonilla, Caroline A. Breitenberger, Charles J. Daniels, Bobby L. Gaffney, Melinda Harrison, Lee E. Hughes, Rodney A. King, Gregory P. Krukonis, A. Javier Lopez, Kirsten Monsen-Collar, Marie C. Pizzorno, Claire A. Rinehart, SEA-PHAGES Program, PHIRE Program, Amanda K. Staples, Emily L. Stowe, Rebecca A. Garlena, Daniel A. Russell, Steven G. Cresawn, Welkin H. Pope, Graham F. Hatfull

**Affiliations:** a Department of Biology, University of Wisconsin—River Falls, River Falls, Wisconsin, USA; b Department of Biological Sciences, University of Pittsburgh, Pittsburgh, Pennsylvania, USA; c Department of Biology, Baylor University, Waco, Texas, USA; d Department of Biology, Montclair State University, Montclair, New Jersey, USA; e Center for Life Sciences Education, The Ohio State University, Columbus, Ohio, USA; f Department of Biological Sciences, University of North Texas, Denton, Texas, USA; g Department of Biological Sciences, Carnegie Mellon University, Pittsburgh, Pennsylvania, USA; h Department of Biology, Western Kentucky University, Bowling Green, Kentucky, USA; i Department of Science, Cabrini University, Radnor, Pennsylvania, USA; j Biology Department, Bucknell University, Lewisburg, Pennsylvania, USA; k Department of Biology, James Madison University, Harrisonburg, Virginia, USA

## Abstract

We report here the complete genome sequences of 44 phages infecting *Arthrobacter* sp. strain ATCC 21022. These phages have double-stranded DNA genomes with sizes ranging from 15,680 to 70,707 bp and G+C contents from 45.1% to 68.5%. All three tail types (belonging to the families *Siphoviridae*, *Myoviridae*, and *Podoviridae*) are represented.

## GENOME ANNOUNCEMENT

Bacteriophages are the most abundant biological entities in the biosphere and represent a large reservoir of undiscovered genetic information (1). Genome comparisons of 46 phages infecting a single *Arthrobacter* host revealed extensive genetic diversity (2), with phage clusters distinctly separated from phages isolated on *Mycobacterium smegmatis* (3). Here, we report the genome sequences of 44 additional phages isolated on *Arthrobacter* sp. strain ATCC 21022 (4). These phages were recovered from filtered soil samples by either direct plating (phages Temper16 and Wheelbite) or enrichment (the other 42 phages) and originate from diverse geographical locations (Table 1). Electron microscopy showed that 30 of the phages belong to the family *Siphoviridae*, 7 with prolate heads (cluster AM) and 23 with isometric capsids (Table 1). Eleven phages belong to the family *Myoviridae* and three to the family *Podoviridae* (Table 1).

**TABLE 1:** Properties of 44 *Arthrobacter* phages

Phage name	Cluster	GenBank accession no.	Genome length (bp)	G+C content (%)	No. of CDSs^a^	Genome end type^b^	Virion tail morphology	Virion head morphology	Location of isolation
Canowicakte	AK	MF140400	43,914	61.2	62	13-base 3′ ext.	*Siphoviridae*	Isometric	Radnor, PA
Christian	AK	MF140404	43,081	60.7	61	13-base 3′ ext.	*Siphoviridae*	Isometric	West Deptford, NJ
Dino	AK	MF140407	43,562	61.1	62	13-base 3′ ext.	*Siphoviridae*	Isometric	Lewisburg, PA
Greenhouse	AK	KX688103	43,977	60.8	61	13-base 3′ ext.	*Siphoviridae*	Isometric	Denton, TX
Huntingdon	AK	MG210949	43,891	60.7	61	13-base 3′ ext.	*Siphoviridae*	Isometric	Huntingdon Valley, PA
Lucy	AK	KX576641	42,944	60.7	60	13-base 3′ ext.	*Siphoviridae*	Isometric	Immaculata, PA
MeganNoll	AK	MG198782	44,258	60.9	62	13-base 3′ ext.	*Siphoviridae*	Isometric	Pittsburgh, PA
Nubia	AK	MF140424	44,045	60.7	61	13-base 3′ ext.	*Siphoviridae*	Isometric	Waco, TX
Oxynfrius	AK	KX688102	44,163	60.8	62	13-base 3′ ext.	*Siphoviridae*	Isometric	Denton, TX
PitaDog	AK	MF140425	42,963	60.7	61	13-base 3′ ext.	*Siphoviridae*	Isometric	New Castle, DE
RcigaStruga	AK	KX576640	43,891	60.7	62	13-base 3′ ext.	*Siphoviridae*	Isometric	Radnor, PA
Suppi	AK	KX621004	43,914	61.2	62	13-base 3′ ext.	*Siphoviridae*	Isometric	Mays Landing, NJ
Temper16	AK	MF668285	43,950	61.9	60	13-base 3′ ext.	*Siphoviridae*	Isometric	Columbus, OH
Urla	AK	MG198779	43,940	61	62	13-base 3′ ext.	*Siphoviridae*	Isometric	Pittsburgh, PA
Vallejo	AK	KX621005	43,607	61	62	13-base 3′ ext.	*Siphoviridae*	Isometric	Radnor, PA
LiSara	AL	MF140418	60,137	64.7	96	Circ. perm.	*Siphoviridae*	Isometric	Lewisburg, PA
Shrooms	AL	MF140427	59,707	64.5	98	Circ. perm.	*Siphoviridae*	Isometric	Aquilla, TX
Wheelbite	AL	MF140434	59,879	64.8	94	Circ. perm.	*Siphoviridae*	Isometric	Argyle, TX
Arcadia	AM	MF189170	58,059	45.2	97	9-base 3′ ext.	*Siphoviridae*	Prolate	Radnor, PA
Cheesy	AM	MF324906	58,739	45.2	101	9-base 3′ ext.	*Siphoviridae*	Prolate	River Falls, WI
Correa	AM	MF189171	57,401	45.2	96	9-base 3′ ext.	*Siphoviridae*	Prolate	Radnor, PA
Elsa	AM	MF189172	58,059	45.2	97	9-base 3′ ext.	*Siphoviridae*	Prolate	Exton, PA
Heisenberger	AM	MF189173	58,208	45.1	100	9-base 3′ ext.	*Siphoviridae*	Prolate	Owensboro, KY
Nason	AM	MF189174	58,059	45.1	97	9-base 3′ ext.	*Siphoviridae*	Prolate	Radnor, PA
Tribby	AM	MF189175	59,084	45.2	102	9-base 3′ ext.	*Siphoviridae*	Prolate	Lewisburg, PA
Swenson	AN	MF140429	15,680	59.9	26	11-base 3′ ext.	*Siphoviridae*	Isometric	Pittsburgh, PA
Molivia	AQ	MF185731	58,247	53.5	98	1,467-bp DTR	*Siphoviridae*	Isometric	Radnor, PA
Chocolat	AR	KX670787	69,798	61.7	111	Circ. perm.	*Myoviridae*	Isometric	Philadelphia, PA
Chubster	AR	KX670786	70,258	61.7	112	Circ. perm.	*Myoviridae*	Isometric	Lansdowne, PA
Colucci	AR	MF185718	70,707	61.7	114	Circ. perm.	*Myoviridae*	Isometric	Mississauga, ON
Conboy	AR	KX522650	70,096	61.7	111	Circ. perm.	*Myoviridae*	Isometric	Radnor, PA
EdgarPoe	AR	KX855961	70,176	61.6	111	Circ. perm.	*Myoviridae*	Isometric	Philadelphia, PA
HumptyDumpty	AR	KX855962	69,978	61.7	111	Circ. perm.	*Myoviridae*	Isometric	Radnor, PA
JayCookie	AR	MF668274	70,362	61.7	112	Circ. perm.	*Myoviridae*	Isometric	River Falls, WI
Kabreeze	AR	MF185721	70,035	61.7	111	Circ. perm.	*Myoviridae*	Isometric	Radnor, PA
RosiePosie	AR	MF185723	70,396	61.7	112	Circ. perm.	*Myoviridae*	Isometric	Immaculata, PA
Scavito	AR	MF185724	70,123	61.6	112	Circ. perm.	*Myoviridae*	Isometric	West Chester, PA
Tophat	AR	MF185725	70,091	61.6	111	Circ. perm.	*Myoviridae*	Isometric	Lewisburg, PA
Abidatro	AS	MF140397	39,122	68.5	66	12-base 3′ ext.	*Siphoviridae*	Isometric	Montclair, NJ
ElephantMan	AU	MF038791	58,405	49.9	93	9-base 3′ ext.	*Siphoviridae*	Isometric	Columbus, OH
Niktson	AU	MF038790	58,405	49.9	93	9-base 3′ ext.	*Siphoviridae*	Isometric	Columbus, OH
Adat	AV	MF668266	45,428	45.7	56	1,551-bp DTR	*Podoviridae*	Isometric	Columbus, OH
GurgleFerb	AV	MF668273	45,426	45.7	56	1,550-bp DTR	*Podoviridae*	Isometric	Columbus, OH
Nellie	AV	MF668279	45,428	45.8	57	1,551-bp DTR	*Podoviridae*	Isometric	Columbus, OH

a CDSs, protein-coding sequences.

b ext., extension; DTR, direct terminal repeat; Circ. perm., circularly permuted.

Genomic DNA was isolated from plaque-purified phages and sequenced using the Illumina MiSeq platform. The 150-bp single-end reads were assembled into complete genomes using Newbler and Consed, with at least 180-fold coverage. Genome lengths range from 15,680 bp to 70,707 bp, with G+C contents from 45.1% to 68.5% (Table 1), compared to the host genome’s G+C content of 63.4% (4). Thirty of the genomes have defined termini, with either 3′ single-stranded DNA extensions or long (~1.5-kb) direct terminal repeats; the other 14 genomes are circularly permuted, and the first coordinate was assigned near the start of the predicted terminase gene (Table 1). Genomes were annotated using DNAMaster (http://cobamide2.bio.pitt.edu), and coding sequences (between 26 and 114) were predicted using GeneMark (5) and Glimmer (6). Putative protein functions were determined using BLASTp (7) and HHpred (8). Molivia is the only genome with tRNA genes, and six were predicted by Aragorn (9) and tRNAscan-SE (10).

All of the newly isolated phages share sequence similarity to various extents with previously described *Arthrobacter* phages and warrant either inclusion into extant clusters (AK, AL, AM, AN, AQ, AR, and AU) or the formation of new clusters (AS and AV) by joining with previously designated singleton genomes (Table 1). Cluster AS is formed by the similarity of phage Abidatro to the former singleton Galaxy, and they share 93.4% average nucleotide identity spanning ~90% of their genome lengths; both encode integrase and immunity repressor proteins and are likely temperate. The *attP* sites are unusually displaced over 2 kb from the integrase genes, and the intervening region is variable between the two genomes. None of the other phages have genes associated with lysogeny and are predicted to be lytic.

Phages Adat, GurgleFerb, and Nellie share >90% average nucleotide identity spanning ~84% of their genome lengths with Jasmine, forming cluster AV. All of these phages have a podoviral morphology and are the only *Podoviridae* members described among over 5,000 morphologically examined actinobacteriophages (11). However, phages Adat, GurgleFerb, and Nellie lack the putative tail spike protein and lipolytic esterase genes of Jasmine (18 and 19), which are replaced with a putative rhamnogalacturonan acetylesterase gene, perhaps reflecting host range differences.

### Accession number(s).

Nucleotide sequence accession numbers are provided in Table 1.
